# When intelligence begins to act: a thoughtful appraisal of agentic AI in biomedicine

**DOI:** 10.1093/bib/bbag213

**Published:** 2026-05-06

**Authors:** Partha Pratim Ray

**Affiliations:** Sikkim University, Gangtok, India

**Keywords:** LLM, semantic agents, bioinformatics, pervasive biomedical informatics

## Abstract

The expanding role of intelligent systems in biomedical science marks a shift from passive analysis towards active participation in discovery and care. Recent scholarship has begun to frame these systems not merely as models, but as agents capable of planning, interaction, and adaptation. This letter reflects on such developments, acknowledging their conceptual clarity and practical ambition, while raising questions about evaluation, responsibility, human judgement, and long-term scientific culture. The intent is to encourage careful reflection as these technologies move closer to real-world integration.

I read the published review on large language model (LLM) agents in bioinformatics and biomedicine with sustained attention and genuine appreciation [1]. The article is ambitious in scope, yet disciplined in execution. It succeeds where many reviews falter: it does not simply enumerate systems, but constructs a coherent intellectual framework for understanding what it truly means for an AI system to become an ‘agent’ in biomedical contexts.

What is especially commendable is the authors’ insistence on architectural clarity. By grounding agentic behaviour in planning, perception, action, and memory, the review article moves the discussion beyond fashionable terminology and into engineering reality. This framing helps distinguish genuine agentic systems from loosely coupled toolchains, and it gives readers a language to reason about strengths, failure modes, and design trade-offs. In this sense, the review performs an important act of conceptual hygiene for a rapidly evolving field.

The treatment of applications is equally strong. Rather than portraying a single success narrative, the article carefully juxtaposes promise with limitation. Drug discovery, protein engineering, clinical decision support, mental health applications, and multi-omics analysis are all presented as domains of opportunity, but also as domains where errors carry real consequences. The inclusion of comparative limitations-such as lack of wet-lab validation, reliance on static knowledge, or narrow evaluation settings-adds credibility and maturity to the discussion.

I also appreciated the depth with which ethical, legal, and societal concerns are handled. These sections are not appended as an afterthought, but woven into the overall argument. Issues of hallucination, bias, privacy, and legal responsibility are framed not as abstract risks, but as structural challenges inherent to agentic systems operating in sensitive environments. This realism is essential if such systems are to be taken seriously by clinicians, regulators, and patients alike.

That said, the article also prompts several questions that may warrant further dialogue.

First, evaluation remains a central unresolved issue. Many reported gains are measured in terms of task completion, benchmark accuracy, or efficiency. In biomedicine, however, the most critical failures often occur in edge cases, rare conditions, or ambiguous scenarios [2]. How should agentic systems be evaluated for resilience under uncertainty, disagreement between data sources, or incomplete evidence? Without such evaluation regimes, there is a risk of overestimating readiness for real-world deployment.

Second, the discussion of human–agent collaboration is compelling, but it leaves open a fundamental boundary question. When an agent plans workflows, selects tools, and synthesizes conclusions, where does human responsibility truly begin and end? In practice, clinicians and researchers may defer, consciously or unconsciously, to systems that appear confident and systematic [3]. Designing for collaboration may therefore require not only better interfaces, but explicit mechanisms that preserve human doubt, dissent, and override.

Third, the vision of continual learning raises subtle concerns about epistemic stability. Biomedical knowledge is not only cumulative; it is also conservative, built on validation, replication, and guidelines [4]. How can agentic systems update themselves without eroding hard-won consensus or reintroducing disproven hypotheses? A balance between adaptability and institutional memory seems crucial, yet remains technically and organizationally undefined.

Fourth, multi-agent collaboration is presented as a path towards robustness and scalability. While this is persuasive, collective systems can also amplify shared biases or obscure accountability [5]. If a distributed group of agents arrives at a flawed recommendation, how should that error be traced, audited and corrected? Transparency at the system level may prove more challenging than at the single-model level.

Finally, the broader cultural implication deserves attention. As agents increasingly generate hypotheses, design experiments and draft interpretations, the nature of scientific practice itself may shift. There is a risk that human intuition, serendipity, and critical scepticism could gradually give way to procedural efficiency. Preserving these human qualities may be as important as improving model performance.

As a closing remark, this article represents a significant and thoughtful contribution to the literature. It offers not only a map of current capabilities, but a lens through which to think responsibly about what comes next. The questions raised here are offered in the same spirit as the review itself: constructive, forward-looking, and grounded in the belief that the future of biomedical intelligence will be strongest when technical innovation is matched by conceptual care.

**Key Points**

- Agentic AI in biomedicine represents a conceptual shift from passive analysis to systems that plan, act, and adapt within scientific and clinical workflows.
- Clear architectural definitions are essential to distinguish genuine agentic systems from loosely integrated tool-based pipelines.
- Current evaluation practices remain insufficient for biomedical deployment, particularly in uncertain, rare, or high-stakes scenarios.
- Human–agent collaboration must be designed to preserve responsibility, critical judgement, and the ability to dissent or override automated reasoning.
- As agentic systems mature, safeguarding scientific culture, epistemic stability, and accountability will be as important as improving technical performance.

## Data Availability

No data is available.
